# Computational Bioprospecting Guggulsterone against ADP Ribose Phosphatase of SARS-CoV-2

**DOI:** 10.3390/molecules27238287

**Published:** 2022-11-28

**Authors:** Mateusz Kciuk, Somdutt Mujwar, Isha Rani, Kavita Munjal, Adrianna Gielecińska, Renata Kontek, Kamal Shah

**Affiliations:** 1 Department of Molecular Biotechnology and Genetics, University of Lodz, 90-237 Lodz, Poland; 2 Doctoral School of Exact and Natural Sciences, University of Lodz, 90-237 Lodz, Poland; 3 Chitkara College of Pharmacy, Chitkara University, Chandigarh 140401, Punjab, India; 4 Spurthy College of Pharmacy, Marasur Gate, Bengaluru 562106, Karnataka, India; 5 Department of Pharmacognosy, M.M. College of Pharmacy, Maharishi Markandeshwar (Deemed to Be University) Mullana, Ambala 133207, Haryana, India; 6 Institute of Pharmaceutical Research, GLA University, Mathura 281406, Uttar Pradesh, India

**Keywords:** SARS-CoV-2, drug repurposing, ADP ribose phosphatase, COVID-19, Corona, antiviral

## Abstract

Coronavirus Disease-2019 (COVID-19) is a highly contagious disease caused by Severe Acute Respiratory Syndrome-Coronavirus-2 (SARS-CoV-2). The World Health Organization (WHO) classified the disease a as global public health hazard on 11 March 2020. Currently, there are no adequate measures to combat viral infections, including COVID-19, and the medication guidelines for the management of COVID-19 are dependent on previous findings from SARS-CoV and MERS-CoV research. Natural products have achieved widespread acceptance around the world as a means of enhancing healthcare and disease prevention. Plants are a potential source of antiviral factors such as flavonoids, phenolic acids, terpenoids, and others. Some of these agents exhibit a broad spectrum of antiviral activity. This study aimed to screen herbal leads for possible inhibitors of the SARS-CoV-2 ADP Ribose Phosphatase enzyme (ARP). Guggulsterone was found to be highly stabilized within the active site of the viral ARP enzyme by molecular dynamic simulation with very little fluctuation throughout the simulation timeframe of 100 ns. Thus, guggulsterone can be further used to develop a safe and competent medication for evolving therapy against SARS-CoV-2 in post-preclinical and clinical trials.

## 1. Introduction

The basic components of a virus’s structural framework include the protein coat, nucleic acid, viral enzymes, and sometimes, a lipid envelope. This simple structure forces the virus to use the host’s cellular machinery for replication. Therefore, viruses are often referred to as obligatory intracellular pathogens. The development of therapeutics with specific toxicity against viruses is proven more challenging given these properties. Moreover, an effective antiviral drug must specifically block the multiplication of viruses and not damage the infected cell as well as neighboring cells [1,2,3,4,5,6]. Furthermore, drugs can be designed to target viral particles situated on the surface of virions that facilitate the adsorption and entrance of the virus into the host cell or can be optimized to target viral nucleic acids (either DNA or RNA depending on the type of the virus), viral proteins, and viral sheaths formed from the host cell membranes. Antiviral medications, in contrast to the majority of antibiotics, do not kill their target pathogens but prevent them from replicating [7,8,9,10].

The development of new antiviral drugs is a challenging endeavor given the continuous co-evolution of the pathogen and the host. Changes in the genetic makeup of the virus force changes in cell physiology and vice versa. Therefore, viral variations constitute a major burden in antiviral therapy and account for the accelerated design of new antiviral drugs. Furthermore, the introduction of a new antiviral compound for human use is complex, time-consuming, and involves multiple steps, including target identification, screening, lead generation and optimization, clinical studies, and drug registration [9]. Additionally, drug repurposing (repositioning) can be used to repurpose existing medications for use in new therapeutic indications. Phytochemicals derived from medicinal plants constitute a group of well-studied compounds with antiviral properties that can be employed as virus-targeting agents or can be used to design new antiviral compounds [9,10]. Three years after the global coronavirus outbreak, many nations are still trying to encourage citizens to get vaccinated against the disease despite widespread vaccine skepticism and false information [11,12,13,14].

**Figure 1:**
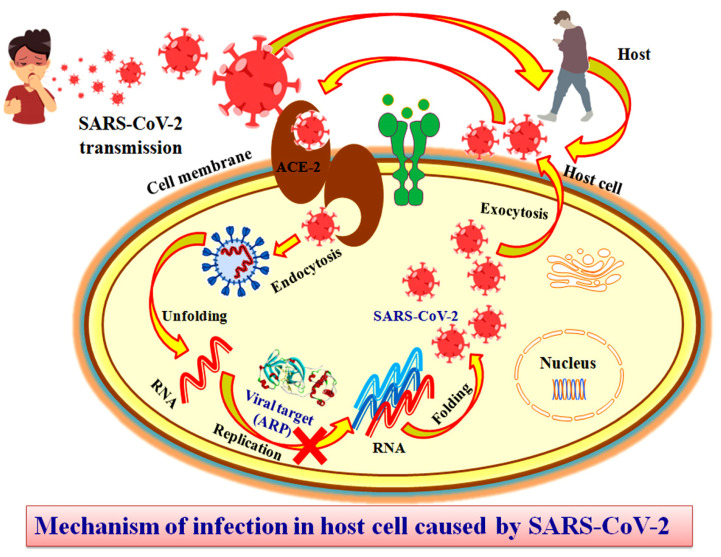
In silico framework for herbal-based screening to recognize viral ARP inhibitors to develop new antiviral therapies for COVID-19. COVID-19 is an airborne disease spread by droplets released during coughing and sneezing. The SARS-CoV-2 virus enters the cell via the angiotensin-converting enzyme 2 (ACE2) through endocytosis. Unfolding of the virus leads to release of the RNA molecule. ADP ribose phosphatase (ARP) encoded by the viral RNA molecule plays an important role in RNA replication as it removes ADP-ribose from ADP-ribosylated proteins and RNA. The inhibition of ARP (possibly with herbal leads identified in this study) may prevent replication of the virus and consecutive steps of SARS-CoV-2 folding and release from the host cell.

SARS-CoV-2 has a single-stranded RNA as the nuclear material coding for structural as well as non-structural proteins [15,16]. The structural proteins of the virus include the spike (S), protein envelope (E), membrane (M), and nucleocapsid (N) proteins, which are encoded by the 5’UTR region of the viral RNA together with a replicase complex. In contrast, viral functional proteins, such as proteases, phosphatases, and polymerases, are encoded by the 3′UTR region that has numerous unidentified ORFs (open reading frames). The ADP ribose phosphatase (ARP) enzyme is one of the most significant metabolic enzymes of SARS-CoV-2, playing a critical role in the virus’s reproduction. ADP-ribose-1 monophosphate (AMP) and its hydrolysis products have been associated with regulatory functions in pathogenic assembly [16,17,18,19,20]. ARP catalyzes the conversion of ADP-ribose to ribose-5-phosphate and AMP. Its inhibition results in the prevention of viral replication. Thus, ARP is considered to be an important antiviral drug target to terminate viral multiplication. To examine, integrate, and regulate considerable information produced from experimental data, in silico approaches are used. Computational drug screening provides a systematic and rational approach for the evaluation of therapeutic possibilities within traditional medications [21,22,23,24]. In this study, an integrated molecular docking simulation-based screening of herbal compounds was performed to identify potential leading compounds that could inhibit the SARS-CoV-2 ARP enzyme and possibly prevent its replication. The rationale behind this study is presented in Figure 1.

## 2. Experimental

### 2.1. Selection and Preparation of Target Protein 

A structural model of ADP ribose phosphatase (ARP) of SARS-CoV-2 associated with its substrate, ADP ribose, was downloaded from the protein databank (PDB code: 6w02) [25,26]. Structural model of the ARP enzyme complexed with ADP ribose is shown in Figure S1. The complexed ligand was detached from the macromolecular complex using Chimera software [27]. The macromolecule was prepared for docking by removing non-reacting water molecules and insertion of polar hydrogen atoms. Rotatable, non-rotatable, and unrotatable bonds were assigned in the ligand (ADP ribose) required for the execution of docking simulations [28,29,30,31,32].

### 2.2. Binding Site Identification

The active ligand site present in the macromolecular target was confirmed using PyMol software and the interaction of target residues with the ligand was observed. The recognized active site in the structural model of the ARP enzyme was further employed to finalize grid parameters for the execution of docking studies [33,34,35,36,37]. Three-dimensional grid covering the active site of viral ARP enzyme was shown in Figure S2.

### 2.3. Molecular Docking

The generated grid-box was utilized by Autogrid to create maps for various sorts of atoms of the ligand and receptor. Autogrid generated A, OA, HD, NA, C, N, SA, and P maps in the current trial. AutoDock software used the provided map files to execute the molecular docking simulations [38,39,40,41,42,43]. 

AutoDock was used as the preferred molecular docking software in the current research. The program utilizes the Lamarckian genetic algorithm (LGA) as its primary conformational search algorithm to perform docking studies. LGA generates a trail population of a range of conformations of the ligand, followed by the generation of mutational conformations and exchange of different parameters, resulting in consecutive generations of biological evolutions to ultimately select individuals with the minimum binding energy. The additional feature of the “Lamarckian” aspect includes the individual and a selective conformational search for its local conformational space, followed by the identifying local minima. The generated information is transferred to the later generations. A semi-empirical force field was utilized to predict the ligand’s binding energy concerning the specific macromolecular target. The force field predicts the binding energy of the ligand by allowing the integration of intra-molecular energies through evaluation of the energies for their bound and unbound states based on a comprehensive thermodynamic model [44,45,46]. The parameters required for docking of the ligand molecule were saved in a file named the docking parameter file [38]. 

### 2.4. Validation of Docking Methodology

The probable binding pattern of the ligand was determined based on its location and alignments, as determined by docking simulations at a molecular level. Parameters contained within the ongoing computational investigation were confirmed by the docking of the ARP enzyme against the reference ADP ribose molecule. The in silico docking process was confirmed by examining the following criteria [24]. 

(a) Overlay Methods

The docking protocols incorporated in the ongoing research work were validated using ligand overlay methodology. The docked conformation should be perfectly inlaid over the crystallized reference conformation of the ligand [22]. 

(b) Chemical Resemblance

If the docked ligand exhibited similar binding interactions with the active residues of the target protein as those found in the crystallized complex, the docking process was successfully validated [23].

### 2.5. In-Silico Screening

A molecular library of 150 diverse ligand molecules from herbal sources was prepared by considering plants traditionally used in the treatment of viral infections and related disorders. Traditional plants considered in the current study included *Allium sativum*, *Camellia sinusis*, *Zinger officinale*, *Nigella sativa*, *Punica granatum*, *Curcuma longa*, *Glycyrrhiza glabra*, *Taraxacum*, *Thymus vulgaris*, *Ocimum tenuiflorum*, *Phyllanthus emblica* Linn., *Trigonella foenum*, *Piper nigrum*, etc. The above-mentioned plants have been reported for the presence of alkaloids, terpenoids, saponins, steroids, glycosides, carbohydrates, monosaccharides, and soluble starch [45,46]. Thus, the prepared ligand library was employed to perform virtual screening with the ARP enzyme, with the intent to identify possible leads. The screening results were further compared with a reported SARS-CoV-2 ARP inhibitor (ZINC82673).

### 2.6. Analysis of Docking Results

Biomolecular interactions for each ligand with the binding residues were considered while evaluating the outcome for each of the ligands against the ARP enzyme. Each ligand’s free binding energy should fall within the predetermined range of −5 to −15 kcal/mol. The compounds having the lowest free energy were shortlisted as lead molecules. The LG algorithm was used as a scoring function with the AutoDock tool [28,38]. The affinity of a certain ligand against a target receptor was calculated using the following mathematical equation.

K_i_ = e^[(ΔG/(RT)]^

where, ΔG = free energy change in binding, T = temperature, and R = gas constant.

### 2.7. Molecular Dynamic Simulation

The thermodynamic stability of the selected leads obtained by virtual screening was analyzed using the Desmond tool in Schrödinger software to conduct molecular dynamics (MD) for 10 ns. Guggulsterone, mahimbine, and andrographolide complex were assessed with the viral ARP enzyme. The molecular dynamic simulation was magnified to 100 ns for the ligand guggulsterone, based on the stability within the macromolecular complex observed in the MD simulation for a shorter period of 10 ns. The TIP3P explicit water model was used to create an orthorhombic-shaped simulation box with a 10 Å gap between the wall of the box and the ligand-protein complex [11,20,24,43,45,46,47,48]. The simulation box’s isosmotic environment was created by adding counter ions with 0.15 M NaCl to neutralize the existing charge. The system’s energy was minimized by running 2000 iterations using a 1 kcal/mol merging threshold. Using an energy-minimized complex system, an MD simulation for 100 ns was executed. A steady 300 K temperature with 1.013 bars of pressure was maintained throughout the simulation process. To construct simulation interaction graphs after the simulation process, the trajectory path was defined as 9.6 with an energy interval of 1.2 ps [11,20,24,43,45,46,47,48]. 

## 3. Results 

### 3.1. Selection and Preparation of Target Protein 

The X-ray diffraction method was used to obtain a three-dimensional protein structural model of the ARP enzyme complexed with the ligand ADP ribose with a resolution of 1.50. The protein structure comprised two comparable chains of 170 amino acids. Using Chimera software, the bound ligand ADP ribose was detached from the complex. The processed macromolecular model was stored in the default AutoDock format. The bound ADP ribose had five aromatic carbons with nine rotatable linkages. In the current study, all 9 bonds of the ADP ribose were preserved as rotatable.

### 3.2. Binding Site Identification 

The binding site of the ARP enzyme was identified during the molecular docking process of the macromolecular target with ADP ribose. Asp22, Ile23, Val49, Ala154, Gly130, Phe132, Asn40, Gly46, Lys44, Gly48, Ala50, Ser128, and Ile131 were found to interact with the native ligand. This information was used for the generation of an imaginary grid-box for further studies [49,50]. Table 1 shows the grid coordinates used to create the grid box, and the docking outcome of ADP ribose against ARP enzyme is tabulated in Table 2.

### 3.3. Validation of Docking Methodology

The following parameters were used to validate the docking approach for executing the docking of the ARP enzyme.

(a) Overlay Method

**Figure 2:**
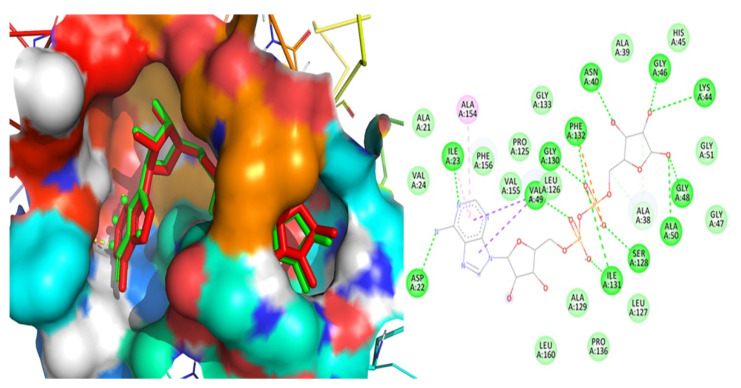
Superimposed conformation of the docked ligand ADP overlaid with its crystallized bioactive conformation and the two-dimensional chemical interaction with the ARP enzyme.

The docked conformation of ADP ribose was impeccably overlapped in its crystallized conformation, signifying the successful validation of the applied docking methodology. The overlay of the docked conformation of the ligand laid over its crystallized conformation as well as the two-dimensional interactions of the reference ligand were shown in Figure 2.

(b) Chemical Resemblance

The docking approach was successfully verified since the docked ligand had identical chemical interactions with the macromolecular residues as those observed for the bioactive crystalline structure of the enzyme [31,46,47,48].

### 3.4. Virtual Screening

**Figure 3:**
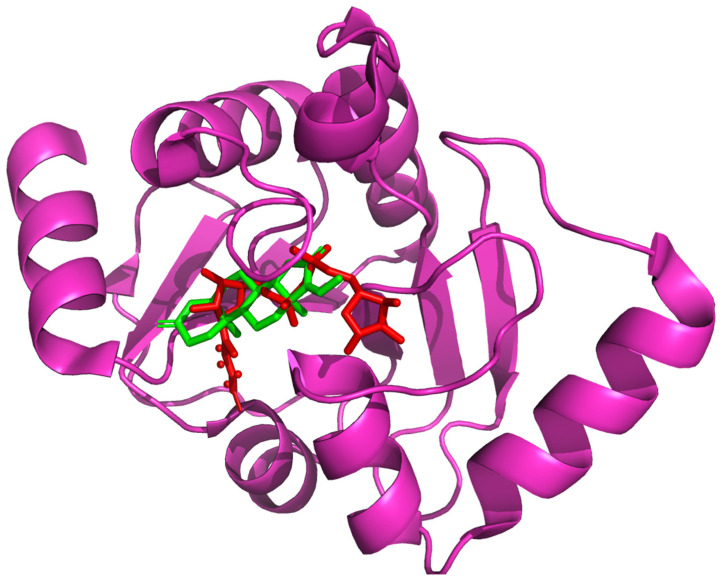
Comparative binding pose of the docked ligand guggulsterone (green) to the native pose of the reference crystallized ligand ADP ribose (red) within the active binding site of the viral ARP enzyme.

After execution of computational screening, the leads were shortlisted based on their ability to bind to the viral ARP. The lowermost free binding energy attained for the most preeminent pose for each ligand and their biomolecular interactions were utilized to calculate their affinity. Table 3 illustrates the free binding energy attained from the in silico screening of the shortlisted lead compounds. Comparative analysis of the obtained screening results with the reported viral ARP inhibitor ZINC82673 showed that the screened herbal lead guggulsterone had a much higher binding affinity for the target receptor (ARP). The comparative binding pose of guggulsterone with respect to the native pose of the reference crystallized ligand (ADP ribose) is shown in Figure 3.

### 3.5. Molecular Dynamic Simulation

**Figure 4:**
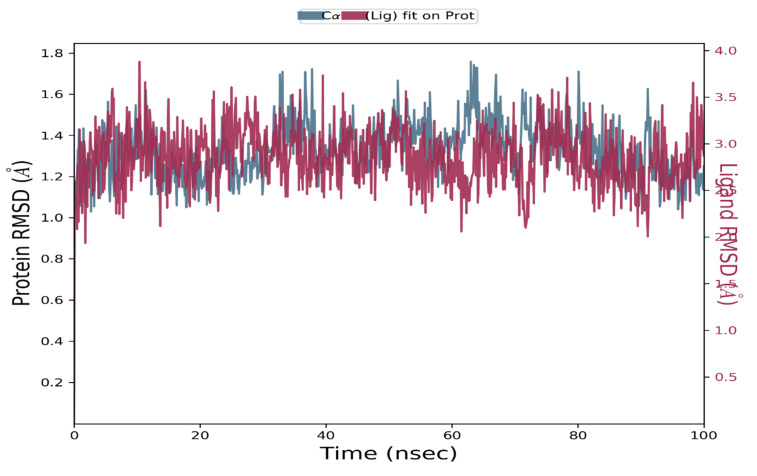
RMSD values of the mutated S protein and complexed guggulsterone recorded during the MD simulation of 100 ns.

**Figure 5:**
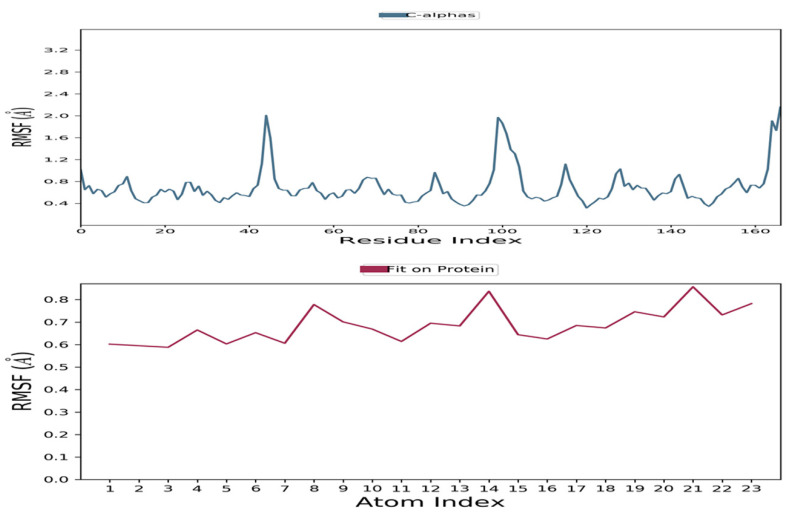
RMSF values of the monomeric subunit of the viral ARP protein of SARS-CoV-2 and complexed guggulsterone recorded during the MD simulation for 100 ns.

Guggulsterone was proposed as a potential antagonist of the viral ARP enzyme of SARS-CoV-2 using dynamic simulation (timeframe of 100 ns) with the help of Desmond tool in Schrodinger software. Based on the atomic selection, the RMSD values for the macromolecular residues were calculated by realigning all macromolecular frames over the stationary reference frame of the backbone in MD simulations. RMSD analysis confirmed the effective operation of the equilibrated simulation process based on structural validation through the procedure. The ligand RMSD indicated the ligand’s stability concerning the macromolecular binding residues throughout the simulation by aligning the heavy metals of the macromolecular binding residues. Throughout the simulation, macromolecular RMSD values remained constant within the range of 1–1.6, confirming that most macromolecular residues were stable enough from their starting state through complexation with the ligand. The stability of the complex ligand within the macromolecular cavity was likewise shown to be within the acceptable range of 2.5–3.5. The RMSD values of both the ligand and the macromolecule remained within the measured range throughout the simulation, indicating successful and stable ligand binding in the macromolecular cavity. Guggulsterone was found to be stabilized within the active site of the viral ARP protein throughout the simulation time. The RMSD values of the protein and ligand detected during the 100 ns simulation are represented in Figure 4. Similarly, the RMSD values of the macromolecular residues were well within the permissible range of 0.4–2.0, with an average of 0.8 [24]. Only a few residues were observed to fluctuate somewhat outside of the pre-established RMSF value; otherwise, most of the ligand residues had fewer changes within the range of 0.5–1. Figure 5 shows a monomeric component of the viral ARP complexed with guggulsterone during a 100 ns MD simulation.

**Figure 6:**
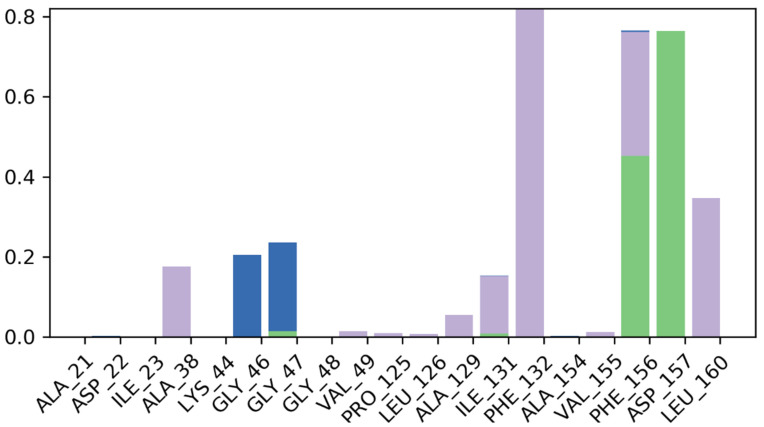
Detailed interactions detected between the ligand guggulsterone with the viral ARP enzyme during 100 ns MD simulation. Hydrophobic interactions are represented in purple; ionic interactions are represented in pink; hydrogen bonds are represented in green; and water bridges are represented in blue.

During the simulation phase, SSE analysis indicated that the macromolecular structure had a total of 47.58% SSE, with 28.58% alpha-helices and 19.01% beta-sheets remaining preserved during the simulation. During the majority of the simulation time, Ala38, Phe132, Leu160, Gly46, Gly47, Phe156, Asp157, and Ile131 interacted with the ligand via hydrogen bonds, hydrophobic interactions, or via a water-bridge, according to the macromolecular complex interaction analysis. More than 5 amino acids were constantly reacting with the complexed guggulsterone during the simulation. Figure 6 depicts the precise macromolecular interactions with the complexed ligand. The RMSD value of the complexed guggulsterone was well below the permissible range of 0.15–0.45, suggesting that guggulsterone oscillated the least during the simulation. The guggulsterone rGyr value was in the range of 3.65–3.80. The MolSA of the ligand was found to be within a range of 292–300 Å2 with an average value of 296 Å2 during the whole simulation process. The SASA of the ligand was between 40–120 Å2 after some fluctuations. The PSA for the complexed guggulsterone was within the range of 86–90 Å2 throughout the simulation.

## 4. Discussion

SARS-CoV-2 is an extremely dangerous pathogen with rapid multiplication potential and an infectious nature that resulted in the outbreak of the COVID-19 global pandemic in 2020. Due to the lack of a specific cure or effective vaccination for the eradication of SARS-CoV-2, the devastating impact of COVID-19 has increased even further. The discovery of multiple selective targets critical for viral replication and spread has contributed to progress in the development of potential medications for COVID-19 disease. The viral ARP enzyme has been revealed to play a key role in viral pathogen proliferation in the host cell and could be potentially utilized as a potential therapeutic target for COVID-19 treatment. As a result, computational screening of clinically approved medications against the viral ARP enzyme would be extremely beneficial for the prompt development of innovative antiviral agents [11,15,20].

The drug development process is extremely complex, time-consuming, and associated with significant financial costs. Given the time constraints connected with the rapid spread of the disease and its deadly nature, the best option for controlling the catastrophic COVID-19 pandemic is to investigate clinically approved medications. The study of molecular interactions between pivotal viral target proteins critical for the virus’s infectious nature and repurposed, clinically-approved medications may increase their application potential [39]. In silico drug repurposing can be utilized to identify and establish new therapeutic applications of existing drugs [11,19,20,21,22]. Drug repurposing and repositioning approaches are now frequently used to develop successful novel medicines for various conditions using drugs already used in the clinic. Computational methodologies such as structure-based drug design can be used to improve the efficiency and accuracy of drug discovery [8,39,49,50].

In the current study, the RCSB protein data repository was used to construct a three-dimensional model of the ARP enzyme complexed with ADP ribose [17,26]. The isolated ligand was redocked with the macromolecular target using AutoDock to validate the settings for the docking procedure. In silico screening led to the identification of lead candidates, such as guggulsterone, mahanimbine, linarin, withanolide, andrographolide, artemisinin, mahanine, silybin, quercetin, luteolin, etc., as potent inhibitors against the SARS-CoV-2 ARP enzyme.

These compounds have been reported in medicinal plants, including *Commiphora wightii* (guggulsterone), *Murraya koenigii* (mahanimbine), *Valeriana officinalis* (linarin), *Withania somnifera* (withanolide), *Andrographis paniculata* (andrographolide), *Artemisia annua* (artemisinin), *M, koenigii* (mahanine), *Silybum marianum* (silybin), *Torreya nucifera* (quercetin), and *Garcinia indica* (luteolin) [51,52,53,54,55,56,57]. Hence, medicinal plants containing these bioactive compounds could constitute effective supplementary treatments for the management to SARS-CoV-2. Moreover, in today’s era of functional foods, the production of vitamin-biofortified foods has significance, and plants are without a doubt the richest providers of numerous vitamins. According to current research, vitamin supplementation has been found to lessen the incidence, severity, and risk of death from pneumonia caused by the cytokine storm of numerous viral infections, including COVID-19 [58,59,60]. The potent health effects of *C. wightii* and its therapeutic constituent, guggulsterone, against atherosclerosis, rheumatism, obesity, and hyper-cholesterolemia have been reported since ancient times [61]. Brobst et al. [62] reported the safety profile of guggulsterone through a cytochrome CYP3A enzyme interaction study. Guggulsterone exerts its biological activity through activation of the alpha isoform of the estrogen receptor, the progesterone receptor, and the pregnane X-receptor. It was shown that guggulsterone-mediated pregnane X receptor activation increased CYP3A gene expression in both rodent and human hepatocytes. Herbal drug interactions are known to occur when the pregnane X receptor is activated; therefore, these findings suggest that guggulsterone treatment should be taken with caution in individuals treated with drugs metabolized by CYP3A family members [62,63,64,65].

*M. koenigii* is an edible plant used in Indian traditional medicine commonly known as curry leaf. It has been associated with numerous health benefits and immunomodulatory, anti-amnesic, anti-epileptic, antioxidant, and anti-inflammatory properties. The safety profile of this plant has been well studied through cytochrome P450 (CYP450) enzyme inhibition assays. Human CYP enzymes play a key role in the oxidative metabolism of drugs and in a variety of xenobiotics. Inhibition of CYP enzymes may result in unexpected unfavorable drug interactions as a result of significant changes in the metabolic clearance of co-administered pharmaceuticals. Additionally, there is an increasing concern about the impacts of isolated bioactive constituents, as well as the influence of whole plant extracts, on CYP activity [65]. The inhibitory effects of various plants extracts, including *M. koenigii* extract, were reported by Pandit et al. [65]. This study revealed the higher inhibitory potential of the whole plant extract (half inhibitory concentrations for major CYP enzymes: CYP3A4 (IC_50_ 160.47 ± 5.45 μg/mL), CYP2D6 (IC_50_ 206.63 ± 1.99 μg/mL), CYP2C9 (IC_50_ 156.56 ± 3.77 μg/mL) and CYP1A2 (IC_50_ 129.66 ± 2.40 μg/mL)) compared to that of mahanimbine (CYP3A4 (IC_50_ 186.67 ± 1.87 μg/mL), CYP2D6 (IC_50_ 249.33 ± 1.14 μg/mL), CYP2C9 (IC_50_ 202.01 ± 2.86 μg/mL), and CYP1A2 (IC_50_ 148.57 ± 1.30 μg/mL). Moreover, the study indicated that the examined extracts and components had a modest degree of impact on drug metabolism and exhibited a low chance of causing severe drug interactions. The study also suggested that mahanimbine, a bioactive compound in *M. koenigii*, was safe for use as a complementary medicine [65].

In a previous investigation, ligands from herbal sources were screened against the viral primary viral protease (Mpro) of SARS-CoV-2 to examine their potential inhibitory effect on the enzyme. Enmozhi et al. used in silico analysis to identify andrographolide (a bioactive component of *A. paniculata*) as a potential lead with Mpro inhibitory activity (binding efficiency of −3.094357 kcal/mol) [66]. Similar in silico analysis identified artemisinin as a possible plant-derived lead for COVID-19 treatment. Recently, the artemisinin preparation (ARTIVeda) exhibited a promising safety profile, with minor side effects in COVID-19 patients including mild rash and a moderate increase in blood pressure. Importantly, the addition of ARTIVeda to the standard of care (SOC) speed up the recovery of patients with COVID-19 experiencing mild to moderate symptoms (ClinicalTrials.gov Identifier: NCT05004753) [67,68].

Guggulsterone and mahanimbine, two potent lead compounds, should also be investigated in preclinical and clinical trials against SARS-CoV-2. In our study, molecular dynamic simulation revealed that guggulsterone was highly stabilized within the binding cavity of the viral ARP enzyme with very little or no fluctuation throughout the simulation timeframe of 100 ns.

Isolated guggulsterone, as the major constituent of the *C. gileadensis* extract, exhibited antiviral activity against Dengue virus (DENV) [69], herpes simplex virus type 2 (HSV-2), respiratory syncytial virus type B (RSV-B), coxsackie virus B type 3, and adenovirus type 5 [70]. Guggulsterone was also previously reported in the literature for its potential beneficial effects against COVID-19 disease [71,72]. Preethi et al. [71] mentioned the immunomodulatory role of guggulsterone in COVID-19 associated with obesity via interaction with farnesoid X receptor and nuclear factor-κB receptor. Mishra et al. [72] identified the antiviral potential of some existing anticancer agents, including guggulsterone, against SARS-CoV-2 Mpro using molecular docking and dynamic simulations. The above evidence indicates the potential of guggulsterone for drug repurposing against COVID-19. Given the evidence of guggulsterone safety in clinical trials [73,74,75] this compound constitutes an important lead for future clinical investigations [75].

## 5. Conclusions

In silico screening has proven to be a highly effective, sensible, and rapid method for discovering existing medicinal molecules with therapeutic effectiveness against SARS-CoV-2 ARP enzyme. Here, a herbal-based ligand library consisting of 150 ligand compounds was virtually tested against the viral ARP enzyme. Guggulsterone, mahanimbine, linarin, withonolide, andrographolide, artemisinin, mahanine, silybin, quercetin, and luteolin were identified as possible herbal lead compounds against the ARP enzyme of SARS-CoV-2. Based on its binding interaction and stability within the binding cavity of the macromolecular receptor, guggulsterone is proposed as a safe and effective lead candidate for developing a COVID-19 therapy against the ARP enzyme of SARS-CoV-2.

## Figures and Tables

**Table 1:** Grid coordinates used for docking of ARP enzyme.

Macromolecule	x-Axis	y-Axis	z-Axis	Spacing (Ả)	x-Center	y-Center	z-Center
6w02	40	40	40	0.452	3.501	−5.927	−22.574

**Table 2:** Docking outcome of ADP ribose against ARP enzyme.

Macromolecule	Binding Residues	RMSD	Binding Energy (kcal/mol)
6w02	Asp22, Ile23, Val49, Ala154, Gly130, Phe132, Asn40, Gly46, Lys44, Gly48, Ala50, Ser128, and Ile131	1.32	−9.59

**Table 3:** The binding energies of shortlisted herbal leads to the viral ARP enzyme.

S. No.	Name	Structure	Binding Energy (kcal/mol)
1.	Guggulsterone	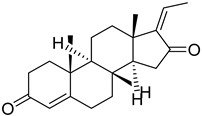	−10.24
2.	Mahanimbine	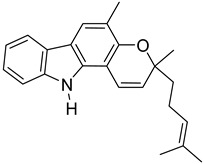	−10.16
3.	Linarin	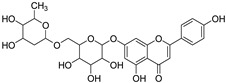	−9.93
4	Withanolide	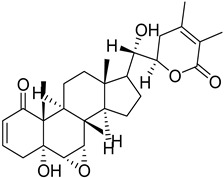	−9.79
5.	Andrographolide	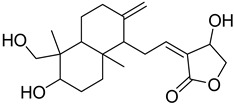	−9.55
6.	Artemisinin	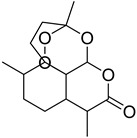	−9.44
7.	Mahanine	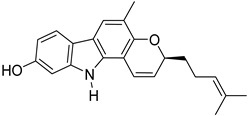	−9.39
8.	Silybin	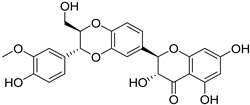	−9.13
9.	Quercetin	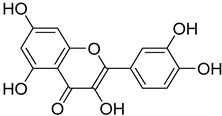	−9.03
10.	Luteolin	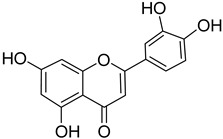	−9.02
11.	ZINC82673	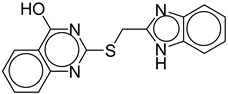	−8.76

## Data Availability

Not applicable.

## Supplementary Materials

The following are available online at https://www.mdpi.com/article/10.3390/molecules27238287/s1, Figure S1: Structural model of the ARP enzyme complexed with ADP ribose, Figure S2: Three-dimensional grid covering the active site of viral ARP enzyme.

## References

[1] Elfiky A.A. (2020). Anti-HCV, nucleotide inhibitors, repurposing against COVID-19. Life Sci..

[2] Elfiky A.A. (2020). Ribavirin, Remdesivir, Sofosbuvir, Galidesivir, and Tenofovir against SARS-CoV-2 RNA dependent RNA polymerase (RdRp): A molecular docking study. Life Sci..

[3] Hoffmann M., Kleine-Weber H., Schroeder S., Krüger N., Herrler T., Erichsen S., Schiergens T.S., Herrler G., Wu N.-H., Nitsche A. (2020). SARS-CoV-2 Cell Entry Depends on ACE2 and TMPRSS2 and Is Blocked by a Clinically Proven Protease Inhibitor. Cell.

[4] Mirza M.U., Froeyen M. (2020). Structural elucidation of SARS-CoV-2 vital proteins: Computational methods reveal potential drug candidates against main protease, Nsp12 polymerase and Nsp13 helicase. J. Pharm. Anal..

[5] Ye Q., Wang B., Zhang T., Xu J., Shang S. (2020). The mechanism and treatment of gastrointestinal symptoms in patients with COVID-19. Am. J. Physiol. Gastrointest. Liver Physiol..

[6] Kumar D., Pandey S.K., Kumar S., Kumar A., Rai D., Munjal K., Rani M., Narayan S. (2020). Change in bone mineral density in premenopausal women with rheumatoid arthritis managed with or without prednisolone. Int. J. Health Clin. Res..

[7] Chen N., Zhou M., Dong X., Qu J., Gong F., Han Y., Qiu Y., Wang J., Liu Y., Wei Y. (2020). Epidemiological and clinical characteristics of 99 cases of 2019 novel coronavirus pneumonia in Wuhan, China: A descriptive study. Lancet.

[8] Sohraby F., Bagheri M., Aryapour H. (2019). Performing an In Silico Repurposing of Existing Drugs by Combining Virtual Screening and Molecular Dynamics Simulation. Methods Mol. Biol..

[9] De Clercq E. (1993). Antiviral agents: Characteristic activity spectrum depending on the molecular target with which they interact. Adv. Virus Res..

[10] Kausar S., Said Khan F., Ishaq Mujeeb Ur Rehman M., Akram M., Riaz M., Rasool G., Hamid Khan A., Saleem I., Shamim S., Malik A. (2021). A review: Mechanism of action of antiviral drugs. Int. J. Immunopathol. Pharm..

[11] Mujwar S. (2021). Computational repurposing of tamibarotene against triple mutant variant of SARS-CoV-2. Comput. Biol. Med..

[12] Darlenski R., Tsankov N. (2020). COVID-19 pandemic and the skin: What should dermatologists know?. Clin. Dermatol..

[13] Kumar A., Mansoori N., Kavitamunjal, Dahiya G., Dwivedi V., Choudhary M. (2019). Comparative efficacy study of a polyherbal formulation with other available drugs in propiobacterium acnes induced rat model. Eur. J. Biomed..

[14] Kciuk M., Gielecińska A., Mujwar S., Mojzych M., Marciniak B., Drozda R., Kontek R. (2022). Targeting carbonic anhydrase IX and XII isoforms with small molecule inhibitors and monoclonal antibodies. J. Enzym. Inhib. Med. Chem..

[15] Jain R., Mujwar S. (2020). Repurposing metocurine as main protease inhibitor to develop novel antiviral therapy for COVID-19. J. Struct. Chem..

[16] Mondal R., Lahiri D., Deb S., Bandyopadhyay D., Shome G., Sarkar S., Paria S.R., Thakurta T.G., Singla P., Biswas S.C. (2020). COVID-19: Are we dealing with a multisystem vasculopathy in disguise of a viral infection?. J. Thromb. Thrombolysis.

[17] Michalska K., Kim Y., Jedrzejczak R., Maltseva N.I., Stols L., Endres M., Joachimiak A. (2020). Crystal structures of SARS-CoV-2 ADP-ribose phosphatase (ADRP): From the apo form to ligand complexes. IUCrJ.

[18] Saikatendu K.S., Joseph J.S., Subramanian V., Clayton T., Griffith M., Moy K., Velasquez J., Neuman B.W., Buchmeier M.J., Stevens R.C. (2005). Structural basis of severe acute respiratory syndrome coronavirus ADP-ribose-1’’-phosphate dephosphorylation by a conserved domain of nsP3. Structure.

[19] Wu C., Liu Y., Yang Y., Zhang P., Zhong W., Wang Y., Wang Q., Xu Y., Li M., Li X. (2020). Analysis of therapeutic targets for SARS-CoV-2 and discovery of potential drugs by computational methods. Acta Pharm. Sin. B.

[20] Mujwar S., Sun L., Fidan O. (2022). In silico evaluation of food-derived carotenoids against SARS-CoV-2 drug targets: Crocin is a promising dietary supplement candidate for COVID-19. J. Food Biochem..

[21] Mujwar S., Deshmukh R., Harwansh R.K., Gupta J.K., Gour A. (2019). Drug Repurposing Approach for Developing Novel Therapy Against Mupirocin-Resistant Staphylococcus aureus. ASSAY Drug Dev. Technol..

[22] Mujwar S., Harwansh R.K. (2022). In Silico Bioprospecting of Taraxerol as a Main Protease Inhibitor of SARS-CoV-2 to Develop Therapy against COVID-19. Struct. Chem..

[23] Mujwar S., Kumar V. (2020). Computational Drug Repurposing Approach to Identify Potential Fatty Acid-Binding Protein-4 Inhibitors to Develop Novel Antiobesity Therapy. ASSAY Drug Dev. Technol..

[24] Mujwar S., Tripathi A. (2021). Repurposing Benzbromarone as Antifolate to Develop Novel Antifungal Therapy for Candida Albicans. J. Mol. Model..

[25] Jin Z., Du X., Xu Y., Deng Y., Liu M., Zhao Y., Zhang B., Li X., Zhang L., Peng C. (2020). Structure of Mpro from COVID-19 virus and discovery of its inhibitors. Nature.

[26] Berman H.M., Westbrook J., Feng Z., Gilliland G., Bhat T.N., Weissig H., Shindyalov I.N., Bourne P.E. (2000). The protein data bank. Nucleic Acids Res..

[27] Pettersen E.F., Goddard T.D., Huang C.C., Couch G.S., Greenblatt D.M., Meng E.C., Ferrin T.E. (2004). UCSF Chimera--a visualization system for exploratory research and analysis. J. Comput. Chem..

[28] Mujwar S., Pardasani K.R. (2015). Prediction of riboswitch as a potential drug target and design of its optimal inhibitors for Mycobacterium tuberculosis. Int. J. Comput. Biol. Drug Des..

[29] Agrawal N., Upadhyay P., Mujwar S., Mishra P. (2020). Analgesic, anti-inflammatory activity and docking study of 2-(substituted phenyl)-3-(naphthalen1-yl)thiazolidin-4-ones. J. Indian Chem. Soc..

[30] Kaur A., Mujwar S., Adlakha N. (2016). In-silico analysis of riboswitch of Nocardia farcinica for design of its inhibitors and pharmacophores. Int. J. Comput. Biol. Drug Des..

[31] Kaushal S.K., Brijendra S., Mujwar S., Prakash B.S. (2019). Molecular Docking based analysis to elucidate the DNA Topoisomerase IIbeta as the potential target for the Ganoderic acid, A natural therapeutic agent in cancer therapy. Curr. Comput. Aided Drug Des..

[32] Minaz N., Razdan R., Hammock B.D., Mujwar S., Goswami S.K. (2019). Impact of diabetes on male sexual function in streptozotocin-induced diabetic rats: Protective role of soluble epoxide hydrolase inhibitor. Biomed. Pharmacother..

[33] DeLano W.L. (2002). Pymol: An open-source molecular graphics tool. CCP4 Newsl. Protein Crystallogr..

[34] Mishra I.M.R., Mujwar S., Chandra P., Sachan N. (2020). A retrospect on antimicrobial potential of thiazole scaffold. J. Heterocycl. Chem..

[35] Mujwar S., Shah K., Gupta J.K., Gour A. (2021). Docking based screening of curcumin derivatives: A novel approach in the inhibition of tubercular DHFR. Int. J. Comput. Biol. Drug Des..

[36] Gupta N., Qayum A., Singh S., Mujwar S., Sangwan P.L. (2022). Isolation, Anticancer Evaluation, Molecular Docking, Drug likeness and ADMET Studies of Secondary Metabolites from Psoralea corylifolia seeds. ChemistrySelect.

[37] Mujwar S., Pardasani K.R. (2015). Prediction of Riboswitch as a potential drug target for infectious diseases: An Insilico case study of anthrax. J. Med. Imaging Health Inform..

[38] Morris G.M., Huey R., Olson A. (2008). Using autodock for ligand–receptor docking. Curr. Protoc. Bioinform..

[39] Mujwar S. (2021). Computational bioprospecting of andrographolide derivatives as potent cyclooxygenase-2 inhibitors. Biomed. Biotechnol. Res. J..

[40] Pradhan P., Soni N.K., Chaudhary L., Mujwar S., Pardasani K.R. (2015). In-silico prediction of riboswitches and design of their potent inhibitors for H1N1, H2N2 and H3N2 strains of influenza virus. Biosci. Biotechnol. Res. Asia.

[41] Shah K., Mujwar S., Gupta J.K., Shrivastava S.K., Mishra P. (2019). Molecular Docking and In Silico Cogitation Validate Mefenamic Acid Prodrugs as Human Cyclooxygenase-2 Inhibitor. ASSAY Drug Dev. Technol..

[42] Shah K., Mujwar S., Krishna G., Gupta J.K. (2020). Computational Design and Biological Depiction of Novel Naproxen Derivative. ASSAY Drug Dev. Technol..

[43] Kciuk M., Mujwar S., Szymanowska A., Marciniak B., Bukowski K., Mojzych M., Kontek R. (2022). Preparation of Novel Pyrazolo [4, 3-e] tetrazolo [1, 5-b][1, 2, 4] triazine Sulfonamides and Their Experimental and Computational Biological Studies. Int. J. Mol. Sci..

[44] Tyagi S., Raghvendra S.U., Kalra T., Munjal K. (2010). Applications of metabolomics-a systematic study of the unique chemical fingerprints: An overview. Int. J. Pharm. Sci. Rev. Res..

[45] Gupta N., Qayum A., Singh S., Mujwar S., Sangwan P.L. (2022). Isolation, Cytotoxicity Evaluation, Docking, ADMET and Drug Likeness Studies of Secondary Metabolites from the Stem Bark of Anthocephalus cadamba (Roxb.). ChemistrySelect.

[46] Agrawal N., Mujwar S., Goyal A., Gupta J.K. (2022). Phytoestrogens as Potential Antiandrogenic Agents Against Prostate Cancer: An In Silico Analysis. Lett. Drug Des. Discov..

[47] Rani I., Kalsi A., Kaur G., Sharma P., Gupta S., Gautam R.K., Chopra H., Bibi S., Ahmad S.U., Singh I. (2022). Surgery, Modern drug discovery applications for the identification of novel candidates for COVID-19 infections. Ann. Med. Surg..

[48] Shinu P., Sharma M., Gupta G.L., Mujwar S., Kandeel M., Kumar M., Nair A.B., Goyal M., Singh P., Attimarad M. (2022). Computational Design, Synthesis, and Pharmacological Evaluation of Naproxen-Guaiacol Chimera for Gastro-Sparing Anti-Inflammatory Response by Selective COX2 Inhibition. Molecules.

[49] Low Z.Y., Farouk I.A., Lal S.K. (2020). Drug Repositioning: New Approaches and Future Prospects for Life-Debilitating Diseases and the COVID-19 Pandemic Outbreak. Viruses.

[50] Panda S., Kumari L., Badwaik H.R., Shanmugarajan D. (2022). Computational Approaches for Drug Repositioning and Repurposing to Combat SARS-CoV-2 Infection. Computational Approaches for Novel Therapeutic and Diagnostic Designing to Mitigate SARS-CoV-2 Infection.

[51] Malik J., Munjal K., Deshmukh R. (2015). Attenuating effect of standardized lyophilized Cinnamomum zeylanicum bark extract against streptozotocin-induced experimental dementia of Alzheimer’s type. J. Basic Clin. Physiol. Pharmacol..

[52] Rose P.W., Prlić A., Altunkaya A., Bi C., Bradley A.R., Christie C.H., Costanzo L.D., Duarte J.M., Dutta S., Feng Z. (2017). The RCSB protein data bank: Integrative view of protein, gene and 3D structural information. Nucleic Acids Res..

[53] Mukherjee P.K., Bahadur S., Harwansh R.K., Biswas S., Banerjee S. (2017). Paradigm shift in natural product research: Traditional medicine inspired approaches. Phytochem. Rev..

[54] Huang F., Li Y., Leung E.L.-H., Liu X., Liu K., Wang Q., Lan Y., Li X., Yu H., Cui L. (2020). A review of therapeutic agents and Chinese herbal medicines against SARS-COV-2 (COVID-19). Pharmacol. Res..

[55] Fernández S., Wasowski C., Paladini A.C., Marder M. (2004). Sedative and sleep-enhancing properties of linarin, a flavonoid-isolated from Valeriana officinalis. Pharmacol. Biochem. Behav..

[56] Munjal K., Ahmad S., Gupta A., Haye A., Amin S., Mir S.R. (2020). Polyphenol-Enriched Fraction and the Compounds Isolated from Garcinia Indica Fruits Ameliorate Obesity through Suppression of Digestive Enzymes and Oxidative Stress. Pharmacogn. Mag..

[57] Singh S., Sharma S., Ali M. (2011). Fatty acid esters from roots of amaranthus hybridus linn. Asian J. Chem..

[58] Meltzer D.O., Best T.J., Zhang H., Vokes T., Arora V., Solway J. (2020). Association of Vitamin D Status and Other Clinical Characteristics With COVID-19 Test Results. JAMA Netw. Open.

[59] Boulkrane M.S., Ilina V., Melchakov R., Fedotova J., Drago F., Gozzo L., Das U.N., Abd El-Aty A.M., Baranenko D. (2020). COVID-19 Disease and Vitamin D: A Mini-Review. Front. Pharmacol..

[60] Munjal K., Sharma S., Sharma S., Kumar D., Choudhary A., Berwal R., Kumar A. (2021). Comparison of serum 25-hydroxyvitamin D levels after a single oral dose of vitamin D3 formulations in mild vitamin D3 deficiency. J. Pharmacol. Pharmacother..

[61] Deng R. (2007). Therapeutic effects of guggul and its constituent guggulsterone: Cardiovascular benefits. Cardiovasc. Drug Rev..

[62] Brobst D.E., Ding X., Creech K.L., Goodwin B., Kelley B., Staudinger J.L. (2004). Guggulsterone activates multiple nuclear receptors and induces CYP3A gene expression through the pregnane X receptor. J. Pharmacol. Exp. Ther..

[63] Zhao M., Ma J., Li M., Zhang Y., Jiang B., Zhao X., Huai C., Shen L., Zhang N., He L. (2021). Cytochrome P450 Enzymes and Drug Metabolism in Humans. Int. J. Mol. Sci..

[64] Fidan O., Mujwar S., Kciuk M. (2022). Discovery of adapalene and dihydrotachysterol as antiviral agents for the Omicron variant of SARS-CoV-2 through computational drug repurposing. Mol. Divers..

[65] Pandit S., Mukherjee P.K., Mukherjee K., Gajbhiye R., Venkatesh M., Ponnusankar S., Bhadra S. (2012). Cytochrome P450 inhibitory potential of selected Indian spices—Possible food drug interaction. Food Res. Int..

[66] Enmozhi S.K., Raja K., Sebastine I., Joseph J. (2021). Andrographolide as a potential inhibitor of SARS-CoV-2 main protease: An in silico approach. J. Biomol. Struct. Dyn..

[67] Tallei T.E., Tumilaar S.G., Niode N.J., Fatimawali, Kepel B.J., Idroes R., Effendi Y., Sakib S.A., Emran T.B. (2020). Potential of Plant Bioactive Compounds as SARS-CoV-2 Main Protease (Mpro) and Spike (S) Glycoprotein Inhibitors: A Molecular Docking Study. Scientifica.

[68] Farmanpour-Kalalagh K., Beyraghdar Kashkooli A., Babaei A., Rezaei A., van der Krol A.R. (2022). Artemisinins in Combating Viral Infections Like SARS-CoV-2, Inflammation and Cancers and Options to Meet Increased Global Demand. Front. Plant Sci..

[69] Chen W.C., Wei C.K., Hossen M., Hsu Y.C., Lee J.C. (2021). (E)-Guggulsterone Inhibits Dengue Virus Replication by Upregulating Antiviral Interferon Responses through the Induction of Heme Oxygenase-1 Expression. Viruses.

[70] Bouslama L., Kouidhi B., Alqurashi Y.M., Chaieb K., Papetti A. (2019). Virucidal Effect of Guggulsterone Isolated from Commiphora gileadensis. Planta Med..

[71] Preethi L., Ganamurali N., Dhanasekaran D., Sabarathinam S. (2021). Therapeutic use of Guggulsterone in COVID-19 induced obesity (COVIBESITY) and significant role in immunomodulatory effect. Obes. Med..

[72] Mishra A., Pathak Y., Choudhir G., Kumar A., Mishra S.K., Tripathi V. (2021). Anticancer natural compounds as potential inhibitors of novel coronavirus (COVID19) main protease: An in-silico study. Cancer Res..

[73] Scholtes C., André P., Trépo C., Cornu C., Remontet L., Ecochard R., Bejan-Angoulvant T., Gueyffier F. (2012). Farnesoid X receptor targeting for hepatitis C: Study protocol for a proof-of-concept trial. Therapie.

[74] Szapary P.O., Wolfe M.L., Bloedon L.T., Cucchiara A.J., DerMarderosian A.H., Cirigliano M.D., Rader D.J. (2003). Guggulipid for the treatment of hypercholesterolemia: A randomized controlled trial. JAMA.

[75] Nohr L.A., Rasmussen L.B., Straand J. (2009). Resin from the mukul myrrh tree, guggul, can it be used for treating hypercholesterolemia? A randomized, controlled study. Complement. Ther. Med..

