# Supplementary material for: Computational Bioprospecting Guggulsterone against ADP Ribose Phosphatase of SARS-CoV-2
Source: Molecules. 2022 Nov 28;27(23):8287. doi: 10.3390/molecules27238287 (PMC9739500; doi:10.3390/molecules27238287)
Supplement: Supplementary file 1 [file molecules-27-08287-s001.zip › molecules-2043260-supplementary.pdf]

Supplementary Data

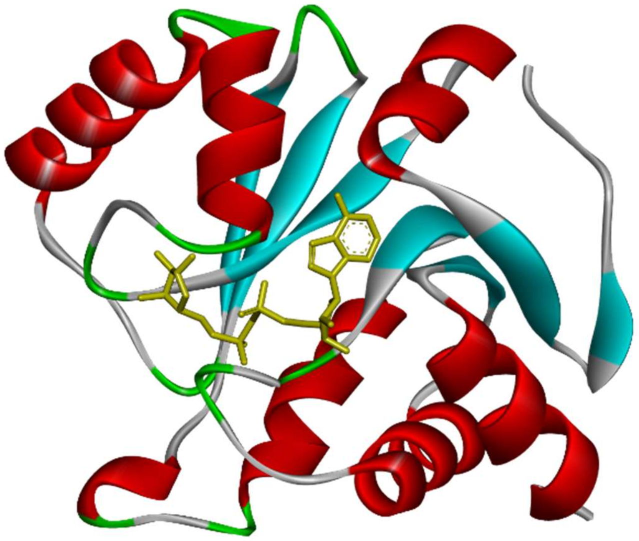

Figure S1. Structural model of the ARP enzyme complexed with ADP ribose.

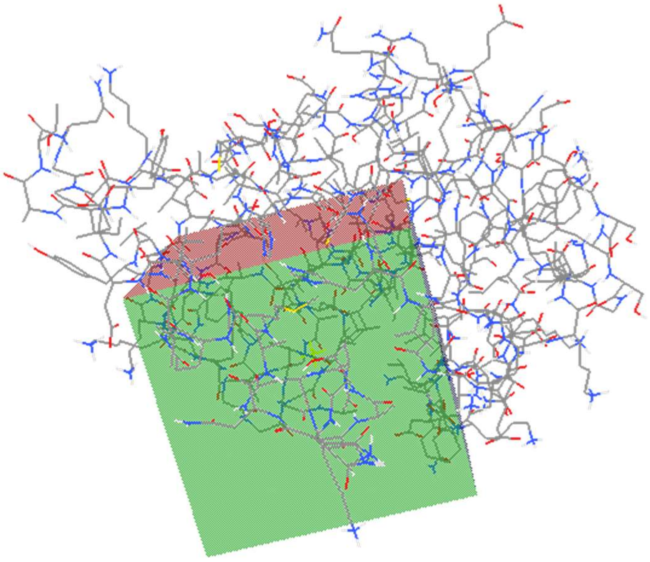

Figure S2. Three-dimensional grid covering the active site of viral ARP enzyme.
